# JOY AND STRESS DURING THE COVID-19 PANDEMIC: FINDINGS FROM THE NATIONAL POLL ON HEALTHY AGING

**DOI:** 10.1093/geroni/igac059.906

**Published:** 2022-12-20

**Authors:** Jessica Finlay, Lindsay Kobayashi, Erica Solway, Dianne Singer, Matthias Kirch, Jeffrey Kullgren, Preeti Malani

**Affiliations:** University of Michigan, Ann Arbor, Michigan, United States; University of Michigan, Ann Arbor, Michigan, United States; University of Michigan, Ann Arbor, Michigan, United States; University of Michigan, Ann Arbor, Michigan, United States; University of Michigan, Ann Arbor, Michigan, United States; University of Michigan, Ann Arbor, Michigan, United States; University of Michigan, Ann Arbor, Michigan, United States

## Abstract

This poll aimed to better understand the complex impacts of the COVID-19 pandemic on the emotional and mental health of older adults. In August 2021, the National Poll on Healthy Aging surveyed a national sample of adults age 50–80 about joys, stresses, and resilience during the pandemic. Most reported feeling a lot (30%) or some (53%) joy these days, while 17% reported feeling very little or no joy. Reports of joy and stress differed substantially by age, sex, physical health, mental health, and household income. For example, people age 50–64 were more likely to report feeling a lot of stress compared with those age 65–80 (25% vs 13%), as were women compared with men (24% vs 15%). The findings may inform public health and policy efforts to support older adults at-risk for poor emotional and mental health, and help cultivate resilience during and after the pandemic.

